# Correction to “Electronic Structure of Few-Layer Black Phosphorus from μ-ARPES”

**DOI:** 10.1021/acs.nanolett.5c00905

**Published:** 2025-04-08

**Authors:** Florian Margot, Simone Lisi, Irène Cucchi, Edoardo Cappelli, Andrew Hunter, Ignacio Gutiérrez-Lezama, KeYuan Ma, Fabian von Rohr, Christophe Berthod, Francesco Petocchi, Samuel Poncé, Nicola Marzari, Marco Gibertini, Anna Tamai, Alberto F. Morpurgo, Felix Baumberger

Our original paper contained two unrelated errors is the tight-binding description which we correct here. Equation 4d of the Supporting Information should read:
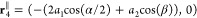
1

In addition, we found an error in the code used for the tight-binding fits of the experimental quasiparticle dispersions. Following the approach in the original publication, we simultaneously fit the corrected tight-binding model to the data of 2*L*, 3*L*, 4*L*, and 5*L* BP along both high-symmetry directions and then calculate as a cross check the dispersion of 9*L* BP, which is not included in the fit. In Figure 1, we overlay the revised global fit and the calculation of the 9*L* dispersion on the ARPES data. The tight-binding parameter values obtained from the revised fit are shown in Table 1.

**Figure 1:**
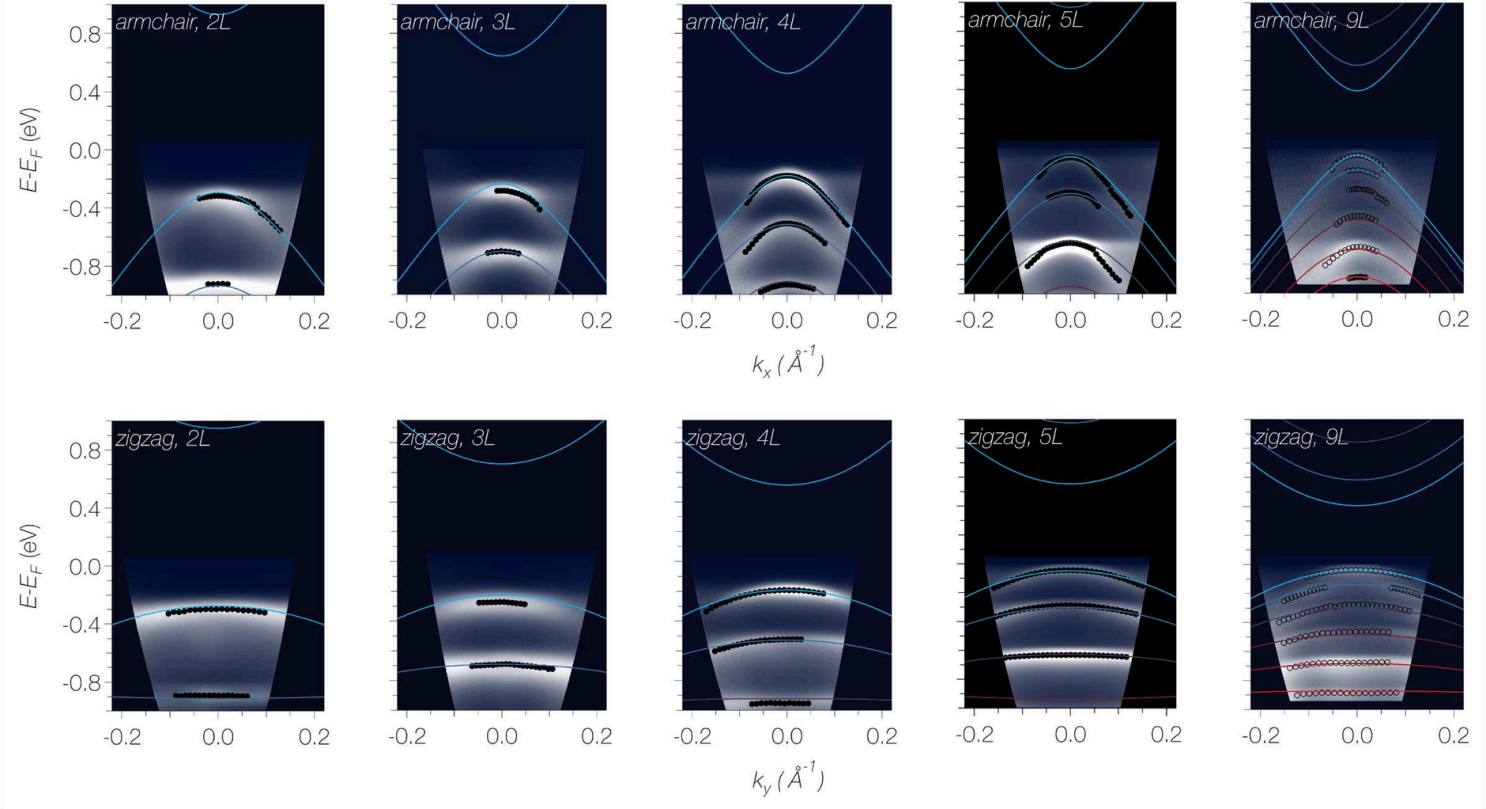
Subband dispersion in ultrathin mechanically exfoliated BP. The experimental ARPES data along the armchair and zigzag direction are shown as false color plots. Solid colored lines are the result of the global tight-binding fit to subband energies extracted from 2*L*, 3*L*, 4*L*, and 5*L* data (filled black circles). The 9*L* data were not included in the fit.

**Table 1:** Revised Tight-Binding Parameters Obtained from a Fit of the Experimental Data

intralayer (eV)		interlayer (eV)	
*t*_1_^∥^	–1.494	*t*_1_^⊥^	0.51
*t*_2_^∥^	3.63	*t*_2_^⊥^	0.27
*t*_3_^∥^	–0.26	*t*_3_^⊥^	–0.087
*t*_4_^∥^	0.364	*t*_4_^⊥^	–0.093

In Figure 2, we compare the anisotropy and thickness dependence of the experimental quasiparticle masses with the revised tight-binding model. This shows a slightly better agreement with the data than found in the original publication.

**Figure 2:**
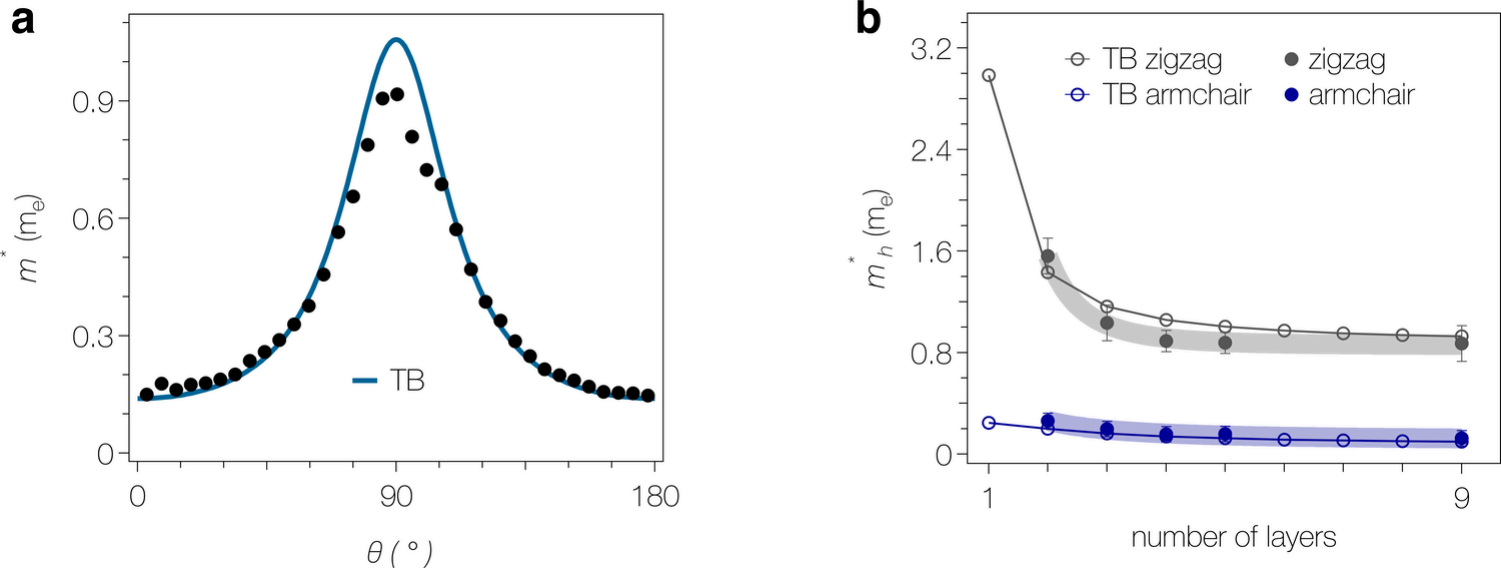
(a) Anisotropy of the effective mass m* extracted from ARPES data together with the angle dependence of m* obtained from the revised tight-binding model. (b) Thickness dependence of m* along both high-symmetry directions. Thick shaded lines are guides to the eye.

The correction of the tight-binding model further affects Figure S2a and b and Figure S3e of the Supporting Information. Revised versions of these figures are available from the authors upon request.

We thank T. Stegmann, Y. Betancour-Ocampo, and J.A. Lizarro Brito for sharing their tight-binding results and for pointing us to the mistake in eq 4d.

This work was supported by the Swiss National Science Foundation (SNSF) Grant 184998.

