# Supplementary material for: Electronic Structure of Few-Layer Black Phosphorus from μ-ARPES
Source: Nano Lett. 2023 Jul 17;23(14):6433–9. doi: 10.1021/acs.nanolett.3c01226 (PMC10375583; doi:10.1021/acs.nanolett.3c01226)
Supplement: Supplementary file 1 — nl3c01226_si_001.pdf [file nl3c01226_si_001.pdf]

Supporting Information: Electronic structure of few-layer black phosphorus from μ-ARPES

Florian Margot,[1] Simone Lisi,[1] Irène Cucchi,[1] Edoardo Cappelli,[1] Andrew Hunter,[1] Ignacio Gutiérrez-Lezama,[1,2] KeYuan Ma,[3] Fabian von Rohr,[1] Christophe Berthod,[1] Francesco Petocchi,[1] Samuel Poncé,[4] Nicola Marzari,[5] Marco Gibertini,[6] Anna Tamai,[1] Alberto F. Morpurgo,[1,2] and Felix Baumberger[1,7]

[1] *Department of Quantum Matter Physics, University of Geneva, 24 quai Ernest Ansermet, CH-1211 Geneva, Switzerland*

[2] *Group of Applied Physics, University of Geneva, 24 quai Ernest Ansermet, CH-1211 Geneva, Switzerland*

[3] *Department of Chemistry, University of Zürich, Winterthurerstrasse 190, CH-8057 Zürich, Switzerland*

[4] *Institute of Condensed Matter and Nanosciences, Université catholique de Louvain, BE-1348 Louvain-la-Neuve, Belgium*

[5] *Laboratory of theory and simulation of materials, École Polytechnique Fédérale de Lausanne, CH-1015 Lausanne, Switzerland*

[6] *Dipartimento di Scienze Fisiche, Informatiche e Matematiche, University of Modena and Reggio Emilia, 41125 Modena, Italy*

[7] *Swiss Light Source, Paul Scherrer Institute, CH-5232 Villigen, Switzerland*

I. SAMPLE FABRICATION

Commercial BP crystals from 2Dsemiconductors were exfoliated on Si/SiO_2 wafers using adhesive tape. Suitable flakes were identified from their contrast in an optical microscope. Heterostructures were prepared with a dry transfer technique using polydimethylsiloxane (PDMS, PF-30/17-X4 from Gel-Pak)/polycarbonate (PC, Merck KGaA) stamps. The graphene encapsulation layer was picked up first and then used to pick up BP and finally the bottom graphite flake that completes the encapsulation. Graphene was picked up with the substrate temperature cycled from 100°C during initial contact to 120°C and back to 100°C for lifting the flake off the substrate. The graphene/BP/graphite stack was released on Au-coated Si/SiO_2 wafers held at 130°C during the initial contact before heating to 180°C (above the glass transition of PC around 150°C) for the transfer from the stamp to the substrate. Immediately after the transfer, the heterostructures were rinsed in CH_3Cl (chloroform) for at least 2 hours. In order to reduce the exposure of BP to reactive gases the entire heterostructure fabrication was performed in a N_2 filled glovebox within $\sim$ 1 h of the exfoliation of BP. Oxygen and water contamination in the glove box were typically below 0.5 ppm. The as-prepared heterostructures were transported under glovebox atmosphere to the ARPES system using a custom built suitcase, which was pumped down to ultra-high vacuum (UHV) pressure without the samples being exposed to ambient atmosphere at any time. Prior to the measurements, the samples were annealed at 200°C for a few minutes, which may remove adsorbants and agglomerate the contaminants trapped in between the 2D materials.

The ARPES measurements on bulk BP were performed on single crystals grown with a modified chemical vapor transport method using a mineralizer as reaction promoter [1]. Specifically, a mixture of 300 mg red phosphorus (99.999%), 20 mg Tin powder (99.995%), and 10 mg Tin(IV)iodide powder (99.998%) were weighed and sealed in an evacuated quartz tube with a length of about 10 cm. The tube was placed horizontally in the center of a box furnace. The temperature of the furnace was increased from room temperature to 650°C at a rate of 1°C/min and held at this temperature for 4 hours. Subsequently, the temperature was decreased to 520°C within 30 hours and was kept at this temperature for 4 hours. Afterwards, the sample was cooled to room temperature over 10 hours. The black phosphorus single crystals were collected from the quartz tube in a glove box. Residual iodine was removed with absolute ethanol, and the crystals were dried in N_2 flow.

II. ARPES MEASUREMENTS

The ARPES measurements of few layer BP were performed in a custom built micro-ARPES system [2]. This setup is based on a continuous wave laser delivering up to 10^{15} photons/s in a μeV bandwidth at a wavelength of 206 nm (6.01 eV). The laser beam is first expanded and then focused with an aberration corrected lens mounted in UHV on a 3-axes translator. The samples are mounted on a conventional 6-axes ARPES manipulator and scanned with a stepper-motor driven, bellow sealed xyz-stage. An overall spatial resolution < 2 μm was determined from knife edge scans. ARPES experiments are performed with a hemispherical analyzer (MB Scientific) equipped with a deflector lens permitting the acquisition of 2D k-space maps without rotating the sample. The typical energy and momentum resolution of the measured photoelectrons was 0.003 Å^{-1} and 5 meV. The experiments used linear polarization and a photon flux of approximately 10^{14} s^{-1} (≈ 100 μW), which is around 2 orders of magnitude higher than typical photon fluxes used at synchrotron based micro-ARPES beamlines based on capillary optic focusing. All micro-ARPES data were acquired at a pressure $p < 10^{-10}$ mbar and a manipulator temperature of $T \sim 4.5$ K.

The ARPES measurements of bulk BP were performed at the SIS beamline of the Swiss Light Source using circularly polarized light with energies of $20-40$ eV. Bulk crystals were cleaved in UHV at a temperature of 10 K, but measured at 50 K in order to avoid charging.

III. TIGHT-BINDING MODEL OF FEW LAYER BLACK PHOSPHORUS

We follow Ref [3] and describe the band structure of few-layer black phosphorus with an effective single orbital tight-binding model with the Hamiltonian:

$$\mathcal{H} = \sum_{ij,\sigma} t^{\parallel}_{ij} c^{\dagger}_{i,\sigma} c_{j,\sigma} + \sum_{ij,\sigma} t^{\perp}_{ij} c^{\dagger}_{i,\sigma} c_{j,\sigma} + \phi \sum_{i,\sigma} c^{\dagger}_{i\sigma} c_{i,\sigma} \tag{1}$$

Here, $t^{\parallel}_{ij}$ and $t^{\perp}_{ij}$ are the intralayer and interlayer hopping parameters between sites i and j, respectively, and ϕ is a chemical potential fixing parameter. Monolayer BP has four basis atoms which we label A, B, C, D, as illustrated in Fig. S 1. Denoting the operators creating fermions on them by $a^{\dagger}_{\alpha}$,$b^{\dagger}_{\alpha}$,$c^{\dagger}_{\alpha}$,$d^{\dagger}_{\alpha}$, we can write the tight-binding

Hamiltonian of a single monolayer as:

$$\mathcal{H}_{1L} = \sum_{\mathbf{k},\sigma} \begin{pmatrix} a^\dagger_{\mathbf{k},\sigma} & b^\dagger_{\mathbf{k},\sigma} & d^\dagger_{\mathbf{k},\sigma} & c^\dagger_{\mathbf{k},\sigma} \end{pmatrix} \underbrace{\begin{pmatrix} H^{\parallel}_{AA}(\mathbf{k}) & H^{\parallel}_{AB}(\mathbf{k}) & H^{\parallel}_{AD}(\mathbf{k}) & H^{\parallel}_{AC}(\mathbf{k}) \\ (H^{\parallel}_{AB}(\mathbf{k}))^* & H^{\parallel}_{AA}(\mathbf{k}) & (H^{\parallel}_{AC}(\mathbf{k}))^* & H^{\parallel}_{AD}(\mathbf{k}) \\ H^{\parallel}_{AD}(\mathbf{k}) & H^{\parallel}_{AC}(\mathbf{k}) & H^{\parallel}_{AA}(\mathbf{k}) & H^{\parallel}_{AB}(\mathbf{k}) \\ (H^{\parallel}_{AC}(\mathbf{k}))^* & H^{\parallel}_{AD}(\mathbf{k}) & (H^{\parallel}_{AB}(\mathbf{k}))^* & H^{\parallel}_{AA}(\mathbf{k}) \end{pmatrix}}_{H_{1L}(\mathbf{k})} \begin{pmatrix} a_{\mathbf{k},\sigma} \\ b_{\mathbf{k},\sigma} \\ d_{\mathbf{k},\sigma} \\ c_{\mathbf{k},\sigma} \end{pmatrix} \tag{2}$$

with the matrix elements:

$$H^{\parallel}_{AA}(\mathbf{k}) = t^{\parallel}_3 \sum_{\mathbf{r}^{\parallel}_3} e^{i\mathbf{k}\cdot\mathbf{r}^{\parallel}_3} \tag{3a}$$

$$H^{\parallel}_{AB}(\mathbf{k}) = t^{\parallel}_1 \sum_{\mathbf{r}^{\parallel}_1} e^{i\mathbf{k}\cdot\mathbf{r}^{\parallel}_1} \tag{3b}$$

$$H^{\parallel}_{AC}(\mathbf{k}) = t^{\parallel}_2 \sum_{\mathbf{r}^{\parallel}_2} e^{i\mathbf{k}\cdot\mathbf{r}^{\parallel}_2} + t^{\parallel}_4 \sum_{\mathbf{r}^{\parallel}_4} e^{i\mathbf{k}\cdot\mathbf{r}^{\parallel}_4} \tag{3c}$$

$$H^{\parallel}_{AD}(\mathbf{k}) = 0 \tag{3d}$$

The vectors $\mathbf{r}^{\parallel}_i$ represented in Fig. S1, are given by:

$$\mathbf{r}^{\parallel}_1 = (-a_1\cos(\alpha/2), \pm a_1 \sin(\alpha/2)) \tag{4a}$$

$$\mathbf{r}^{\parallel}_2 = (a_2\cos(\beta), 0) \tag{4b}$$

$$\mathbf{r}^{\parallel}_3 = (0, \pm 2\sin(\alpha/2)a_1) \tag{4c}$$

$$\mathbf{r}^{\parallel}_4 = (0, (2a_1\cos(\alpha/2) + a_2\cos(\beta)) \tag{4d}$$

In multilayer BP, sublattices A and B of one layer are coupled to sublattices C and D of the adjacent layer. We describe this coupling with the submatrix:

$$H_{\perp}(\mathbf{k}) \equiv \begin{pmatrix} 0 & 0 & H^{\perp}_{AD}(\mathbf{k}) & H^{\perp}_{AC}(\mathbf{k}) \\ 0 & 0 & (H^{\perp}_{AC}(\mathbf{k}))^* & H^{\perp}_{AD}(\mathbf{k}) \\ 0 & 0 & 0 & 0 \\ 0 & 0 & 0 & 0 \end{pmatrix} \tag{5}$$

with matrix elements:

$$H^{\perp}_{AD}(\mathbf{k}) = t^{\perp}_2 \sum_{\mathbf{r}^{\perp}_2} e^{i\mathbf{k}\cdot\mathbf{r}^{\perp}_2} + t^{\perp}_3 \sum_{\mathbf{r}^{\perp}_3} e^{i\mathbf{k}\cdot\mathbf{r}^{\perp}_3} \tag{6a}$$

$$H^{\perp}_{AC}(\mathbf{k}) = t^{\perp}_1 \sum_{\mathbf{r}^{\perp}_1} e^{i\mathbf{k}\cdot\mathbf{r}^{\perp}_1} + t^{\perp}_4 \sum_{\mathbf{r}^{\perp}_4} e^{i\mathbf{k}\cdot\mathbf{r}^{\perp}_4} \tag{6b}$$

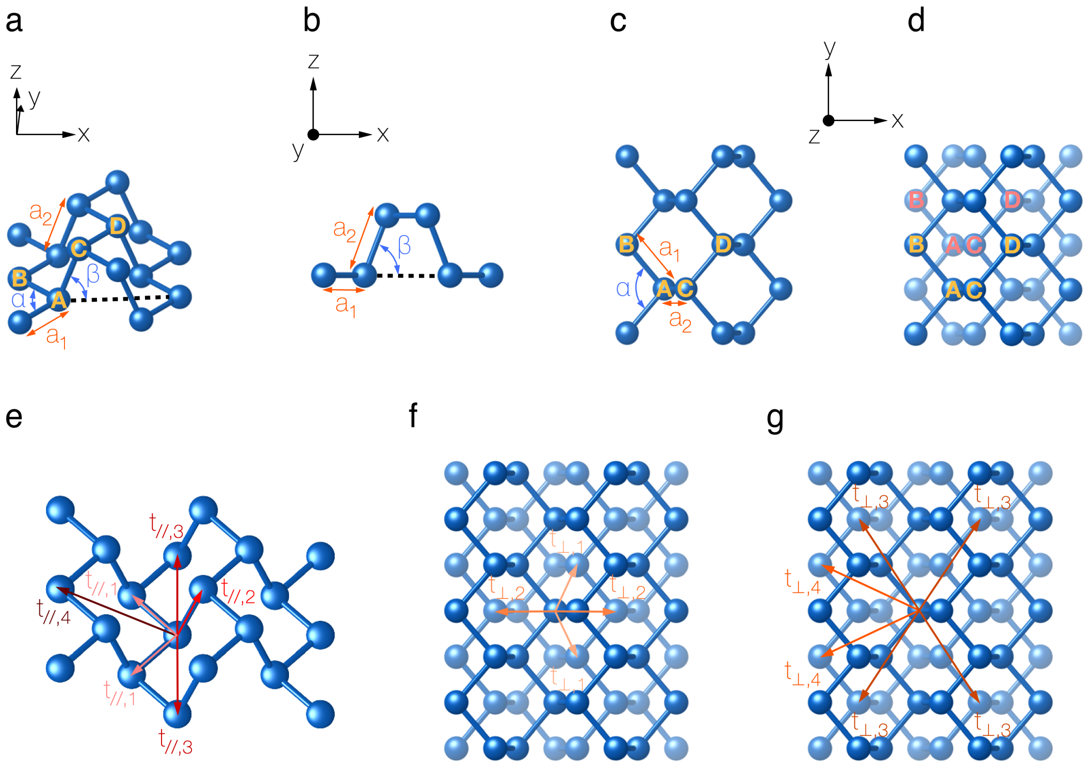

Figure S1. Crystal structure of black phosphorus (BP). a-c) Monolayer BP seen from different angles with the definition of the parameters a_1,a_2, α and β used to describe the vectors $\mathbf{r}_i^{\|}, \mathbf{r}_i^{\perp}$ corresponding to the hopping elements $t_i^{\|}, t_i^{\perp}$ used in the model. A,B,C,D denote the basis sites of individual layers. d) Top view of multilayer BP showing the AB stacking with adjacent layers shifted by half a unit cell in the x direction with respect to each other. e-g) Illustration of the inter- and intralayer hopping parameters $t_i^{\|}, t_i^{\perp}$.

The vectors $\mathbf{r}_i^{\perp}$ are given by:

$$\mathbf{r}_1^{\perp} = (a_1 \cos(\beta), \pm a_1 \sin(\alpha/2)) \tag{7a}$$

$$\mathbf{r}_2^{\perp} = (\pm(a_1 \cos(\alpha/2) + a_2 \cos(\beta)), 0) \tag{7b}$$

$$\mathbf{r}_3^{\perp} = (\pm(a_1 \cos(\alpha/2) + a_2 \cos(\beta)), \pm 2a_1 \sin(\alpha/2)) \tag{7c}$$

$$\mathbf{r}_4^{\perp} = (-(2a_1 \cos(\alpha/2) + a_2 \cos(\beta)), \pm a_1 \sin(\alpha/2)) \tag{7d}$$

The tight-binding Hamiltonian for multilayer BP can now be written as:

$$H_{ML}(\mathbf{k}) = \begin{pmatrix} H_{1L}(\mathbf{k}) & H_\perp(\mathbf{k}) & 0 & 0 & & 0 & 0 & 0 \\ H_\perp^\dagger(\mathbf{k}) & H_{1L}(\mathbf{k}) & H_\perp(\mathbf{k}) & 0 & \dots & 0 & 0 & 0 \\ 0 & H_\perp^\dagger(\mathbf{k}) & H_{1L}(\mathbf{k}) & H_\perp(\mathbf{k}) & & 0 & 0 & 0 \\ & & & & \ddots & & & \\ 0 & 0 & 0 & 0 & \dots & H_\perp^\dagger(\mathbf{k}) & H_{1L}(\mathbf{k}) & H_\perp(\mathbf{k}) \\ 0 & 0 & 0 & 0 & & 0 & H_\perp^\dagger(\mathbf{k}) & H_{1L}(\mathbf{k}) \end{pmatrix} \quad (8)$$

For our analysis of the experimental data, we used the lattice parameters $a_1 = 2.22$ Å, $a_2 = 2.24$ Å, $\alpha = 96.5°$, $\beta = 72°$ and diagonalized the Hamiltonian in Eq. 8 numerically to obtain the tight-binding band structure of few-layer BP. The tight-binding hopping elements were determined by fitting the eigenvalues of Eq. 8 to the experimental band dispersion of $2L$, $3L$, $4L$ and $5L$ BP along both high symmetry directions. The fits were additionally constrained to reproduce a bulk band gap of 400 meV, as it was used in Ref [3] - close to the experimentally determined bulk bandgap (about 300 meV) [4]. With this procedure, we find the hopping parameters shown in TABLE 1 of the main text.

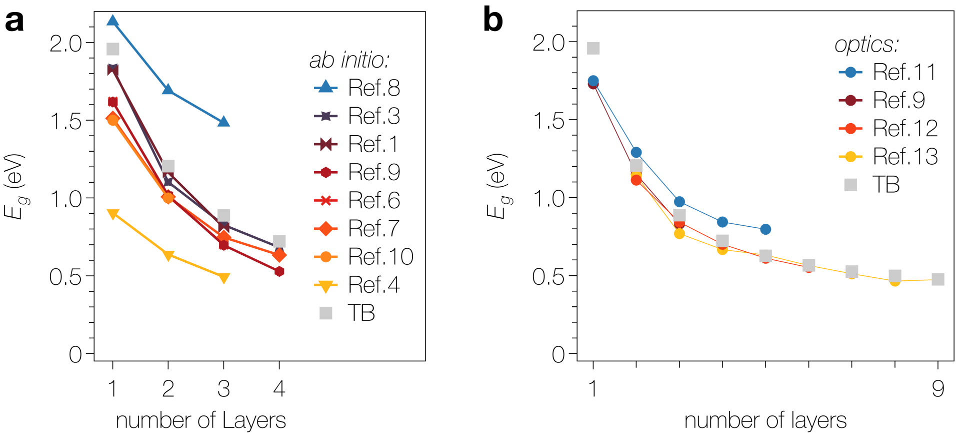

Figure S2. a) Comparison of the electronic gap from our tight-binding model with *ab-initio* electronic structure calculations from Refs. [3, 5–12]. b) Comparison with optical gaps reported by Refs. [11, 13–15]. The TB bands were obtained by constraining the gap of bulk BP to 400 meV.

As shown in Fig. S2, these hopping elements also provide a fair description of measured optical gaps and of quasiparticle gaps from *ab-inito* calculations. Note that the quasiparticle- and optical gap differ by the exciton binding energy. We

speculate that the two gaps might be similar in our devices because the renormalization of the quasiparticle band gap due to dielectric screening in the encapsulation layer [16] might be comparable to the exciton binding energy.

IV. THICKNESS DETERMINATION OF EXFOLIATED BLACK PHOSPHORUS FLAKES

We determine the thickness of BP flakes from a combination of atomic force microscopy (AFM) measurements, the optical contrast on Si/SiO_2 substrates, tight-binding model, *ab-initio* calculations, and our ARPES data, as illustrated in Fig. S3. AFM measurements were performed under ambient conditions in a Cypher AFM from Oxford Instruments using the tapping mode. For $2L-4L$ flakes, we find a nearly linear optical contrast in the red channel of a calibrated microscope. This allows for a rapid determination of the thickness of very thin flakes prior to the encapsulation in graphene/graphite. For thicker flakes, the contrast begins to saturate and we find it more reliable to determine the thickness by comparing measured subband energies with our tight-binding model fitted to $2L-5L$. No $1L$ data is shown throughout the manuscript because we have not succeeded in obtaining high-quality monolayer heterostructures.

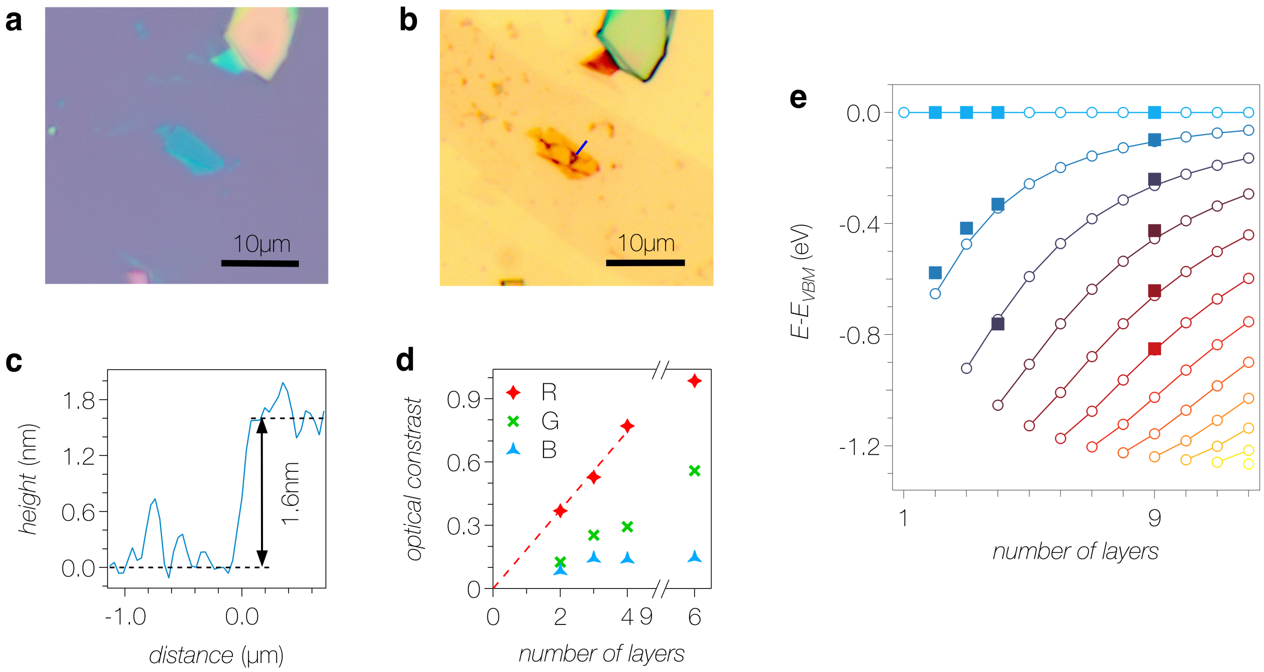

Figure S3. a) Optical micrograph of a BP flake on a Si substrate with 300 nm of thermally grown SiO_2. b) Optical micrograph of the same flake encapsulated with graphene and graphite on a Si/SiO_2 substrate covered with gold. c) AFM line profile obtained on the flake shown in a,b) after encapsulation with graphene. The step height is close to the nominal value of 1.6 nm of trilayer BP [10]. d) Optical contrast obtained in the R,G and B channels. e) Evolution of the sub-band maxima at the Γ point in few layer BP. Hollow dots were obtained from the tight-binding calculations using the hopping parameters given in TABLE 1 of the main text. Filled squares are the QP peak position of the ARPES data at the Γ point.

V. DENSITY-FUNCTIONAL CALCULATION OF FEW LAYER BP

Density functional theory (DFT) calculations have been carried out using the Quantum ESPRESSO distribution [17]. A pseudopotential approach has been adopted to treat electron-ion interactions, with the P ultrasoft pseudopotential chosen from the pslibrary 1.0.0 [18], which yield results in agreement with all electron calculations. The cutoff energy on wavefunctions was set at 40 Ry, and at 400 Ry for the density. To account for van der Waals interactions between the layers the optB88 exchange-correlation functional has been adopted [19], while spurious interactions between artificially periodic replicas of the multilayers along the vertical direction were suppressed using a cutoff on all relevant interactions [20]. The Brillouin zone was sampled with a Γ-centered $10 \times 12 \times 1$ Monkhorst-Pack grid. The atomic structure and unit cell parameters have been fully relaxed until forces on atoms were smaller than 12.5 meV/Å and stresses below 0.5 kbar. We found that the lattice parameters are strongly dependent on the multilayer thickness and rapidly approach the bulk values.

In Fig. S4, we compare our DFT band structure with the subband dispersion extracted from experiment. Note that our DFT calculations for $9L$ BP find a metallic state with two Dirac points along the zigzag high-symmetry line.

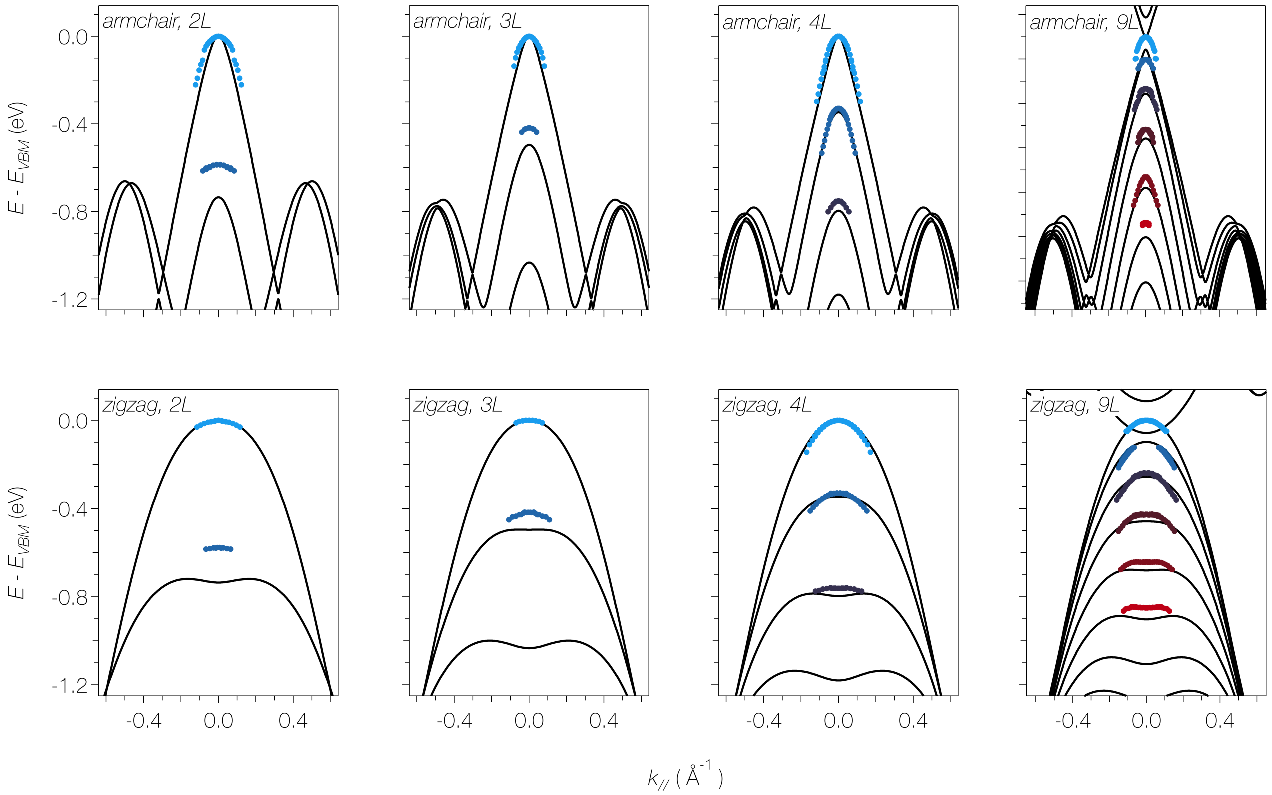

Figure S4. Comparison of the experimental dispersion from the main text with our *ab-initio* calculations. All energies are relative to the valence band edge E_{VBM}. For clarity, the experimental data points have been symmetrized around $k_{\|} = 0$. The colours symbolize the band index.

In Fig. S5, we compile subband energies and effective masses from published theoretical studies and compare them with the values determined from our experiment and *ab-initio* calculations. A significant spread of theoretical subband energies is evident from the figure. We note that the agreement between our *ab-initio* calculations and experiment is good for subbands energies and zigzag effective masses whereas our *ab-initio* effective masses for the armchair direction are over a factor of two smaller than in experiment.

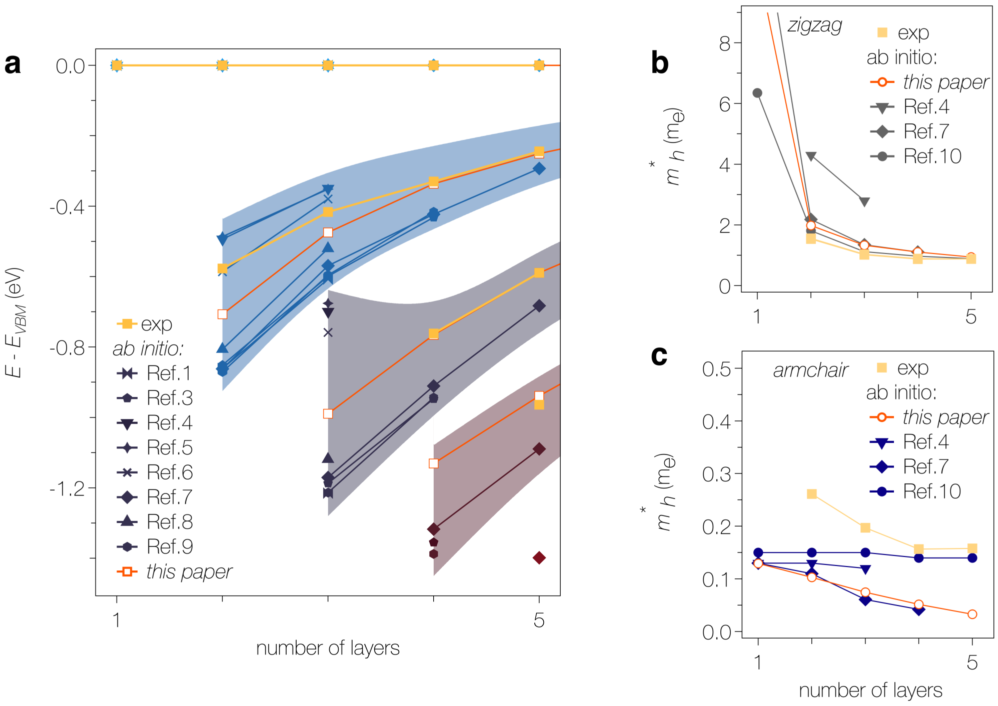

Figure S5. a) Subband energies at the Γ point. Note that all values are given relative to the valence band maximum E_{VBM}. Orange markers represent the experimentally determined subband energies. Theoretical values from Refs. [3, 5–11] are shown in blue, violet and burgundy. The subband energies determined from the *ab initio* calculation of our study are shown as hollow red rectangles. Shaded areas group the different subbands. b-c) Effective masses determined from our experiment and from the electronic structure calculations of Refs. [6, 9, 12].

VI. SPECTRAL FUNCTION OF A FILLED BAND COUPLED TO EINSTEIN PHONONS

At second-order in ionic displacements and neglecting the Debye-Waller correction, electron-phonon coupling is described by the Hamiltonian [21]:

$$\mathcal{H} = \sum_{\mathbf{k},n,\sigma} \xi_{\mathbf{k},n} c^{\dagger}_{n,\mathbf{k},\sigma} c_{n,\mathbf{k},\sigma} + \sum_{\mathbf{q},\nu} \hbar\omega_{\mathbf{q}} (b^{\dagger}_{\mathbf{q},\nu} b_{\mathbf{q},\nu} + 1/2) + \sum_{\mathbf{k},\mathbf{q},m,n,\nu,\sigma} g_{mn\nu}(\mathbf{k},\mathbf{q}) c^{\dagger}_{m,\mathbf{k+q},\sigma} c_{n,\mathbf{k},\sigma} (b^{\dagger}_{\mathbf{q},\nu} + b_{\mathbf{q},\nu}) \quad (9)$$

Here $g_{mn\nu}(\mathbf{k},\mathbf{q})$ are the electron-phonon coupling matrix elements, $\xi_{\mathbf{k},n}$ is the dispersion of band n relative to the chemical potential μ with corresponding creation and annihilation operators $c^{\dagger}_{m,\mathbf{k},\sigma}$, $c^{\dagger}_{m,\mathbf{k},\sigma}$ for each spin orientation

σ. $\omega_{\mathbf{q},\nu}$ is the phonon frequency in branch ν for crystal momentum $\mathbf{q}$ and $b^{\dagger}_{\mathbf{q},\nu}$, $b_{\mathbf{q},\nu}$ are the corresponding phonon creation and annihilation operators.

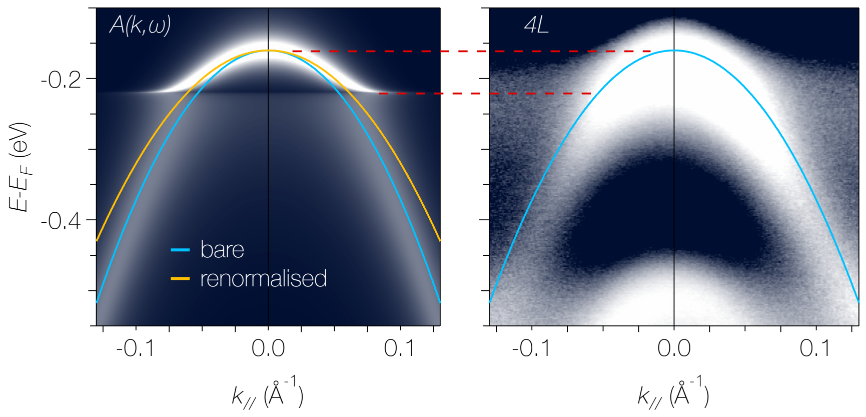

Figure S6. left: Spectral function of an electron-phonon coupled system calculated from Eq. 10 for a mass enhancement $\lambda = 0.32$. The bare and renormalized dispersions are overlaid. Horizontal red lines mark the top of the subband and the phonon frequency $\hbar\Omega = 60$ meV. right: Experimental spectral function from $4L$ BP shown with enhanced contrast. The intense feature around -0.6 eV is the top of the second subband, which is not included in the calculation on the left.

To capture the essence of the spectral function of an electron-phonon coupled system, it is sufficient to consider a single band and a single, dispersionless phonon branch, Ω_0. We further assume that this phonon mode couples to the electrons with a momentum independent electron-phonon matrix element, which we denote by g^2. Using these simplifications, the self-energy Σ is given at second order by:

$$\Sigma(\epsilon) = g^2 \int_{-\infty}^{\infty} \mathrm{d}\xi \, \rho(\xi) \left[\frac{f(\mu - \xi) + b(\hbar\Omega_0)}{\epsilon + i\gamma - \hbar\Omega_0 - \xi} + \frac{f(\xi - \mu) + b(\hbar\Omega_0)}{\epsilon + i\gamma + \hbar\Omega_0 - \xi} \right] \tag{10}$$

where $\rho(\xi)$ is the density of states per spin of the single-particle band considered here, $f(\xi)$ and $b(\hbar\Omega_0)$ are the Fermi-Dirac and the Bose-Einstein distribution, respectively and γ is a broadening factor that can be interpreted as impurity scattering. Note that within this approximation the self-energy is local, *i.e.* it does not dependent on momentum.

From Eq. 10, one obtains for the mass enhancement λ at zero temperature:

$$\lambda = \frac{m^*}{m_{\mathrm{band}}} - 1 = - \left. \frac{\partial \Sigma'(\epsilon)}{\partial \epsilon} \right|_{\epsilon = E_{\mathrm{VBM}}} = \frac{g^2 \rho_0}{\hbar\Omega_0} \tag{11}$$

Here, ρ_0 is the bare density of states per spin at the subband edge. We point out that this expression differs by a factor of 2 from the equivalent expression for metals [21]. This is because the second term in Eq.10 vanishes at zero temperature in our case, contrarily to the case of metals.

Fig. S6 shows the spectral function $A(k,\omega) = -\frac{1}{\pi} \frac{\mathrm{Im}\Sigma(\epsilon)}{[\omega - \xi_k - \mathrm{Re}\Sigma(\epsilon)]^2 + [\mathrm{Im}\Sigma(\epsilon)]^2}$ obtained from this model for a realistic phonon frequency $\hbar\Omega_0 = 60$ meV and a weak to moderate coupling strength $\lambda = 0.32$. The calculation reproduces a

non-dispersive spectral feature at the same energy observed in the experiment. However, the weight of this feature is smaller than in our data and decays more rapidly away from the main band. The same behavior is found within the momentum average approximation of electron-phonon coupling [22]. Stronger effects of electron-phonon coupling on the spectral function are obtained using the momentum average approximation in 1D [22, 23] but this can be traced back to the divergence of the bare density of states at the band edge, which is absent in 2D.

---

[1] Stefan Lange, Peer Schmidt, and Tom Nilges, "Au_3SnP_7@Black Phosphorus: An Easy Access to Black Phosphorus," Inorganic Chemistry **46**, 4028–4035 (2007).

[2] Irène Cucchi, Ignacio Gutiérrez-Lezama, Edoardo Cappelli, Siobhan Mc Keown Walker, Flavio Y. Bruno, Giulia Tenasini, Lin Wang, Nicolas Ubrig, Céline Barreteau, Enrico Giannini, Marco Gibertini, Anna Tamai, Alberto F. Morpurgo, and Felix Baumberger, "Microfocus Laser-Angle-Resolved Photoemission on Encapsulated Mono-, Bi-, and Few-Layer 1T'-WTe_2," Nano Letters **19**, 554–560 (2019).

[3] A. N. Rudenko, Shengjun Yuan, and M. I. Katsnelson, "Toward a realistic description of multilayer black phosphorus: From GW approximation to large-scale tight-binding simulations," Physical Review B **92**, 085419 (2015).

[4] Fengnian Xia, Han Wang, and Yichen Jia, "Rediscovering black phosphorus as an anisotropic layered material for optoelectronics and electronics," Nature Communications **5**, 4458 (2014).

[5] D. J.P. De Sousa, L. V. De Castro, D. R. Da Costa, J. Milton Pereira, and Tony Low, "Multilayered black phosphorus: From a tight-binding to a continuum description," Physical Review B **96**, 155427 (2017).

[6] Tony Low, A. S. Rodin, A. Carvalho, Yongjin Jiang, Han Wang, Fengnian Xia, and A. H. Castro Neto, "Tunable optical properties of multilayer black phosphorus thin films," Physical Review B **90**, 075434 (2014).

[7] Vy Tran, Ryan Soklaski, Yufeng Liang, and Li Yang, "Layer-controlled band gap and anisotropic excitons in few-layer black phosphorus," Physical Review B **89**, 235319 (2014).

[8] Yohannes Abate, Deji Akinwande, Sampath Gamage, Han Wang, Michael Snure, Nirakar Poudel, and Stephen B. Cronin, "Recent progress on stability and passivation of black phosphorus," Advanced Materials **30**, 1704749 (2018).

[9] Yongqing Cai, Gang Zhang, and Yong-Wei Zhang, "Layer-dependent band alignment and work function of few-layer phosphorene," Scientific Reports **4**, 6677 (2014).

[10] Andres Castellanos-Gomez, Leonardo Vicarelli, Elsa Prada, Joshua O Island, K L Narasimha-Acharya, Sofya I Blanter, Dirk J Groenendijk, Michele Buscema, Gary A Steele, J V Alvarez, Henny W Zandbergen, J J Palacios, and Herre S J van der Zant, "Isolation and characterization of few-layer black phosphorus," 2D Materials **1**, 025001 (2014).

[11] Likai Li, Jonghwan Kim, Chenhao Jin, Guo Jun Ye, Diana Y. Qiu, Felipe H. Da Jornada, Zhiwen Shi, Long Chen, Zuocheng Zhang, Fangyuan Yang, Kenji Watanabe, Takashi Taniguchi, Wencai Ren, Steven G. Louie, Xian Hui Chen,

Yuanbo Zhang, and Feng Wang, "Direct observation of the layer-dependent electronic structure in phosphorene," Nature Nanotechnology **12**, 21–25 (2017).

[12] Jingsi Qiao, Xianghua Kong, Zhi Xin Hu, Feng Yang, and Wei Ji, "High-mobility transport anisotropy and linear dichroism in few-layer black phosphorus," Nature Communications **5**, 4475 (2014).

[13] Jiong Yang, Renjing Xu, Jiajie Pei, Ye Win Myint, Fan Wang, Zhu Wang, Shuang Zhang, Zongfu Yu, and Yuerui Lu, "Optical tuning of exciton and trion emissions in monolayer phosphorene," Light: Science and Applications **4**, e312 (2015).

[14] Guowei Zhang, Andrey Chaves, Shenyang Huang, Fanjie Wang, Qiaoxia Xing, Tony Low, and Hugen Yan, "Determination of layer-dependent exciton binding energies in few-layer black phosphorus," Science Advances **4**, eaap9977 (2018).

[15] Guowei Zhang, Shenyang Huang, Andrey Chaves, Chaoyu Song, V. Ongun Özçelik, Tony Low, and Hugen Yan, "Infrared fingerprints of few-layer black phosphorus," Nature Communications **8**, 14071 (2017).

[16] Lutz Waldecker, Archana Raja, Malte Rösner, Christina Steinke, Aaron Bostwick, Roland J. Koch, Chris Jozwiak, Takashi Taniguchi, Kenji Watanabe, Eli Rotenberg, Tim O. Wehling, and Tony F. Heinz, "Rigid band shifts in two-dimensional semiconductors through external dielectric screening," Phys. Rev. Lett. **123**, 206403 (2019).

[17] P Giannozzi, O Andreussi, T Brumme, O Bunau, M Buongiorno Nardelli, M Calandra, R Car, C Cavazzoni, D Ceresoli, M Cococcioni, N Colonna, I Carnimeo, A Dal Corso, S de Gironcoli, P Delugas, R A DiStasio, A Ferretti, A Floris, G Fratesi, G Fugallo, R Gebauer, U Gerstmann, F Giustino, T Gorni, J Jia, M Kawamura, H-Y Ko, A Kokalj, E Küçükbenli, M Lazzeri, M Marsili, N Marzari, F Mauri, N L Nguyen, H-V Nguyen, A Otero de-la Roza, L Paulatto, S Poncé, D Rocca, R Sabatini, B Santra, M Schlipf, A P Seitsonen, A Smogunov, I Timrov, T Thonhauser, P Umari, N Vast, X Wu, and S Baroni, "Advanced capabilities for materials modelling with quantum ESPRESSO," Journal of Physics: Condensed Matter **29**, 465901 (2017).

[18] Andrea Dal Corso, "Pseudopotentials periodic table: From H to Pu," Computational Materials Science **95**, 337–350 (2014).

[19] Jiří Klimeš, David R Bowler, and Angelos Michaelides, "Chemical accuracy for the van der waals density functional," Journal of Physics: Condensed Matter **22**, 022201 (2009).

[20] Thibault Sohier, Matteo Calandra, and Francesco Mauri, "Density functional perturbation theory for gated two-dimensional heterostructures: Theoretical developments and application to flexural phonons in graphene," Phys. Rev. B **96**, 075448 (2017).

[21] Piers Coleman, *Introduction to Many-Body Physics* (Cambridge University Press, 2015).

[22] Glen L. Goodvin and Mona Berciu, "Momentum average approximation for models with electron-phonon coupling dependent on the phonon momentum," Phys. Rev. B **78**, 235120 (2008).

[23] Mingu Kang, Sung Won Jung, Woo Jong Shin, Yeongsup Sohn, Sae Hee Ryu, Timur K. Kim, Moritz Hoesch, and Keun Su Kim, "Holstein polaron in a valley-degenerate two-dimensional semiconductor," Nature Materials **17**, 676–680 (2018).
